# The M-OVIN study: does switching treatment to FSH and / or IUI lead to higher pregnancy rates in a subset of women with world health organization type II anovulation not conceiving after six ovulatory cycles with clomiphene citrate – a randomised controlled trial

**DOI:** 10.1186/1472-6874-13-42

**Published:** 2013-10-25

**Authors:** Marleen J Nahuis, Nienke S Weiss, Fulco van der Veen, Ben Willem J Mol, Peter G Hompes, Jur Oosterhuis, Nils B Lambalk, Jesper MJ Smeenk, Carolien AM Koks, Ron JT van Golde, Joop SE Laven, Ben J Cohlen, Kathrin Fleischer, Angelique J Goverde, Marie H Gerards, Nicole F Klijn, Lizka CM Nekrui, Ilse AJ van Rooij, Diederik A Hoozemans, Madelon van Wely

**Affiliations:** 1 Department of Obstetrics and Gynaecology, Academic Medical Centre, University of Amsterdam, Amsterdam, the Netherlands

## Abstract

**Background:**

Clomiphene citrate (CC) is first line treatment in women with World Health Organization (WHO) type II anovulation and polycystic ovary syndrome (PCOS). Whereas 60% to 85% of these women will ovulate on CC, only about one half will have conceived after six cycles. If women do not conceive, treatment can be continued with gonadotropins or intra-uterine insemination (IUI). At present, it is unclear for how many cycles ovulation induction with CC should be repeated, and when to switch to ovulation induction with gonadotropins and/or IUI.

**Methods/Design:**

We started a multicenter randomised controlled trial in the Netherlands comparing six cycles of CC plus intercourse or six cycles of gonadotrophins plus intercourse or six cycles of CC plus IUI or six cycles of gonadotrophins plus IUI.

Women with WHO type II anovulation who ovulate but did not conceive after six ovulatory cycles of CC with a maximum of 150 mg daily for five days will be included.

Our primary outcome is birth of a healthy child resulting from a pregnancy that was established in the first eight months after randomisation. Secondary outcomes are clinical pregnancy, miscarriage, multiple pregnancy and treatment costs. The analysis will be performed according to the intention to treat principle. Two comparisons will be made, one in which CC is compared to gonadotrophins and one in which the addition of IUI is compared to ovulation induction only. Assuming a live birth rate of 40% after CC, 55% after addition of IUI and 55% after ovulation induction with gonadotrophins, with an alpha of 5% and a power of 80%, we need to recruit 200 women per arm (800 women in total).

An independent Data and Safety Monitoring Committee has criticized the data of the first 150 women and concluded that a sample size re-estimation should be performed after including 320 patients (i.e. 80 per arm).

**Discussion:**

The trial will provide evidence on the most effective, safest and most cost effective treatment in women with WHO type II anovulation who do not conceive after six ovulatory cycles with CC with a maximum of 150 mg daily for five days. This evidence could imply the need for changing our guidelines, which may cause a shift in large practice variation to evidence based primary treatment for these women.

**Trial registration number:**

Netherlands Trial register NTR1449

## Background

There is a lack of evidence on how to best treat women with World Health Organization (WHO) type II anovulation or polycystic ovary syndrome (PCOS) and clomiphene citrate (CC) failure. Women with WHO type II anovulation have absent or irregular ovulation due to hypothalamic-pituitary dysfunction associated with normal levels of endogenous estradiol. The majority of these women can be diagnosed with PCOS. The diagnosis of PCOS is based on at least two of the following three criteria: oligo-ovulation or anovulation, clinical or biochemical evidence of hyperandrogenism, and polycystic ovaries on ultrasound assessment [1].

CC has been used as a first-line ovulation induction agent for over 40 years [2,3]. Systematic reviews and meta-analyses have shown that CC is indeed the best primary treatment option in therapy-naive women with WHO type II anovulation and PCOS [4-8]. Although 60% to 85% of women starting ovulation induction with CC will ovulate, only about one half will have conceived after six cycles [9,10] the so called women with CC-failure.

When anovulatory women ovulate after CC, but do not conceive after six cycles, the clinical problem occurs, that cannot be answered by guidelines. Most trials, randomised or not, have been directed at therapy naïve women, or at women with PCOS that remained anovulatory after CC, also called CC-resistant women [11-18].

For CC-failure, on one hand, women may continue to use CC for another six cycles. On the other hand, they may switch to ovulation induction with gonadotrophins. Both types of ovulation induction may involve intercourse or may be combined with intra-uterine insemination (IUI).

A reason why women should continue CC with regular intercourse is that women do still get pregnant on CC after six treatment cycles, although pregnancy rates per cycle probably drop despite regular ovulation [2,19]. There is consensus that it is best to limit exposure to CC to 12 treatment cycles, as additional cycles may place the woman at increased risk of borderline ovarian tumours [20]. Although it is thus clear that for safety reasons the upper limit of treatment cycles is 12, it is unclear for how many cycles ovulation induction with CC should be repeated from the perspective of effectiveness.

Several studies and one report of an international consensus meeting advise continuation of treatment with gonadotrophins and/or IUI if pregnancy does not occur within six ovulatory cycles [21-24]. This advice is based upon the presumption that lack of conception despite evidence of ovulation may be due to anti-estrogenic effects of CC on the endometrium.

Pregnancy rates with gonadotrophins can be expected to be higher than with CC in women that do not conceive after six ovulatory cycles on CC. The downside is that ovulation induction with gonadotrophins has major disadvantages. It implies daily injections of gonadotrophins combined with concurrent blood and ultrasound monitoring of follicular growth and development. Multifollicular development is not uncommon, despite careful dose adjustment and monitoring, due to the inherent nature of PCOS. Also, injectable gonadotrophins are expensive and the frequent monitoring of follicular growth implies substantial additional cost. Extra costs are generated because treatment cycles often have to be cancelled to minimize the risks of multiple pregnancy and, more rarely, of ovarian hyperstimulation syndrome [25,26].

In IUI motile spermatozoa are concentrated and placed directly into the uterine cavity, in closer proximity to the released oocyt than is the case after vaginal intercourse. There have been no randomised controlled trials that studied the effect of IUI in women undergoing ovulation-induction with CC or gonadotrophins for oligo- or anovulation. Still, IUI is often added to ovulation induction with CC or gonadotrophins. The NICE guidelines actually recommend IUI and prolonged ovulation induction with CC in women who ovulate on CC but do not conceive [27]. IUI is expected to result in higher pregnancy rates than intercourse but requires extra laboratory work and more hospital visits and is therefore much more expensive.

In summary, in women with WHO type II anovulation or PCOS who ovulate on CC but fail to conceive after six ovulatory cycles, prolonged CC or a switch to gonadotrophins, as well as the addition of IUI are treatment options that are often applied in clinical practice. However, data on cost and effectiveness of these options are lacking. We therefore propose to determine which treatment is most cost-effective, thus facilitating a cost-effective and standardised clinical practice in The Netherlands.

## Objective

Our primary objective is to evaluate four treatment regimens in women with WHO type II anovulation that failed to conceive after six ovulatory cycles with CC in terms of singleton live birth. Secondary objectives are to assess the safety and cost-effectiveness for these treatment regimens and to optimise guideline recommendations.

## Methods

### Participating centres

This study is a multicentre randomised controlled trial in The Netherlands and inclusion started in December 2008.

### Inclusion criteria

We will include women with WHO type II anovulation, who have been ovulatory for six cycles on CC treatment, with a maximum of 150 mg daily for five days, but who did not conceive. Ovulation will be assessed by a basal temperature curve, midluteal progesterone (> 16 nmol/l), detection of LH surge or sonography, depending on the local protocol. All women will have at least one patent Fallopian tube, proven by negative Chlamydia antibody titre (CAT), hysterosalpingography (HSG), transvaginal hydrolaparoscopy (THL) or diagnostic laparoscopy combined with tubal testing (DLS and TT), also depending on the local protocol.

### Exclusion criteria

We will not include women below 18 years, women with abnormal prolactin or thyroid-stimulating hormone values, women with intolerable symptoms when treated with CC like hot flashes affecting daily function, headaches, vision changes, or depression and women who remain anovulatory on CC 150 mg daily for five days, the so called CC-resistant women.

### Ethical considerations

Approval for this study was obtained from the Medical Ethical Committee of the Medical Spectrum Twente Enschede and from the Central Committee on Research involving Human Subjects (CCMO), The Netherlands. In women fulfilling the inclusion criteria, written informed consent is obtained before randomisation is carried out.

### Randomisation

Randomisation is performed by accessing a password protected central internet-based randomisation program and is stratified for hospital. The randomisation list is prepared by an independent statistician. A 2x2 factorial design will be used. This will create four groups:

1) CC plus intercourse

2) CC plus IUI

3) gonadotrophins plus intercourse

4) gonadotrophins plus IUI

### Study management

All women participating in the study will undergo a basic fertility work-up including a semen-analysis and endocrinological investigation to rule out hyperprolactinemia and uncorrected thyroid dysfunction. Ovulation is induced with CC. From the third or fifth day until the seventh or ninth day after spontaneous or progesterone induced menstruation women will take CC with a minimum of 50 mg to a maximum of 150 mg a day. During the first six treatment cycles women will undergo CAT, HSG, THL and/or DLS and TT to exclude double sided tubal occlusion.

Women are eligible for the study after six ovulatory cycles proven by a cycle length of less than 35 days, a biphasic basal temperature curve, midluteal progesterone (> 16 nmol/l), detection of LH surge or sonography, depending on the local protocol.

Women eligible for the study will be referred to a research nurse for counselling and randomisation.

### Interventions

**Figure 1:**
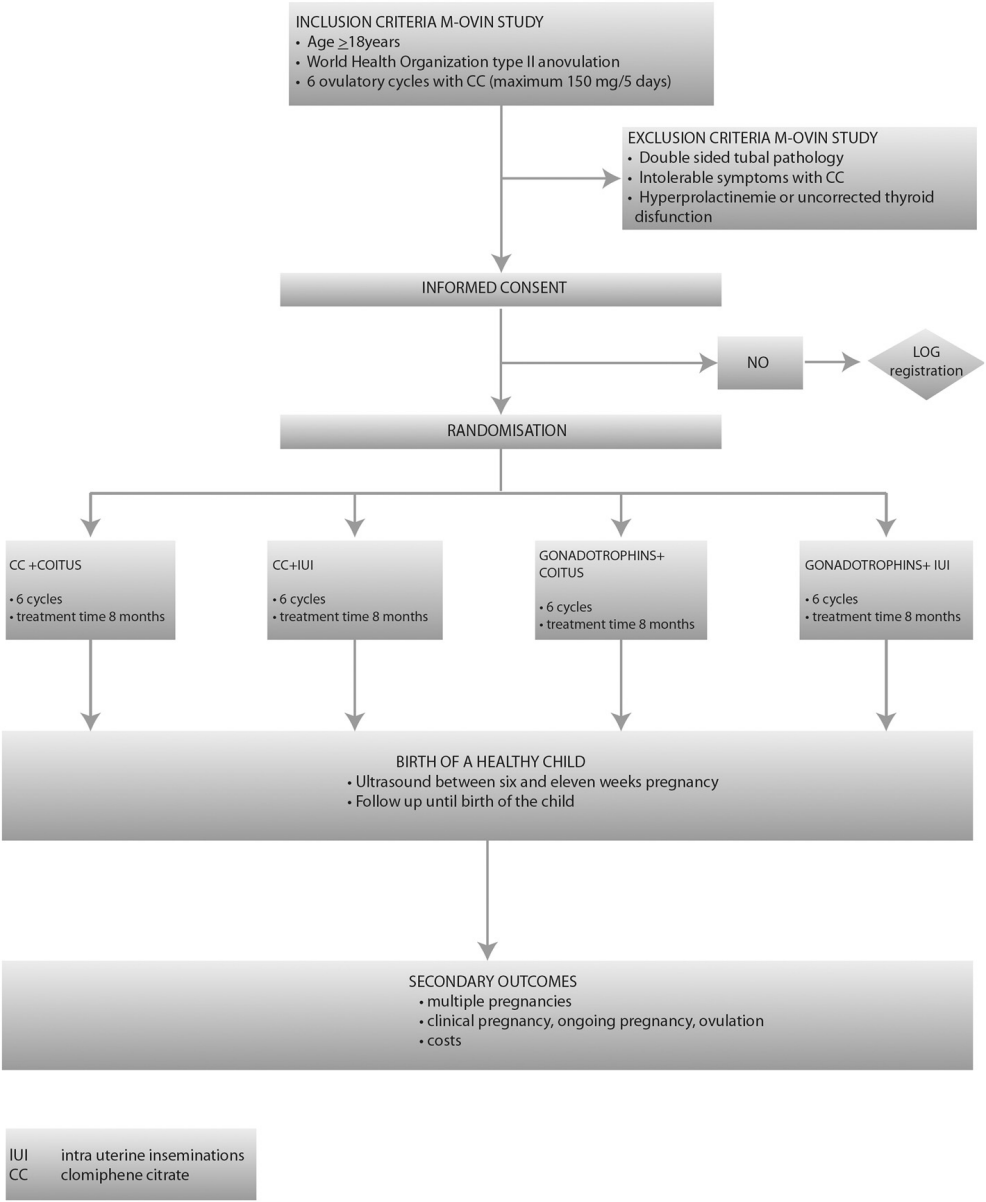
Flow chart M-OVIN study.

Women are allocated to a treatment strategy consisting of six cycles of CC plus intercourse or six cycles of gonadotrophins plus intercourse or six cycles of CC plus IUI or six cycles of gonadotrophins plus IUI. Women are treated until a pregnancy occurs within a treatment time horizon of eight months resulting in the birth of a healthy child (Figure 1).

Ovulation induction, semen preparation and insemination regimens will be done according to hospital specific protocols. Ovulation induction with CC will be started on the third to fifth day of a spontaneous menstrual or progesterone induced bleed, in the same dosage as used in the last ovulatory cycle, with a minimum of 50 mg to a maximum of 150 mg daily, for five days. If ovulation does not occur, the dosage will be increased with steps of 50 mg with a maximum of 150 mg daily in the next cycles. As couples are assumed to have regular intercourse, no specific timing advice will be given. Only if couples have a history of sexual problems they are advised to have regular intercourse, at least three times a week. Ovulation will be documented according to local protocol with either a biphasic temperature curve, or a follicle with a diameter ≥16 mm ;on transvaginal ultrasonography. In case of ovulation women will continue taking the same dose of CC until an end point is reached.

Ovulation induction with gonadotrophins will be started at the second to fifth day of a spontaneous or progesterone induced menstrual bleed according to the established chronic low dose step up regimen. Transvaginal ultrasound will be performed on day three of the cycle or according to local protocol. Women will not be stimulated if they have ovarian cysts >25 mm in mean diameter. The starting dose will be 50 or 75 IU daily. The follicular growth is strictly monitored by transvaginal ultrasound. If more than three dominant follicles (≥18  mm) are present, the cycle will be cancelled. When at least one follicle with a diameter of ≥ 16 mm is present, ovulation is induced by the administration of 5000 IU or 10000 IU of human chorionic gonadotrophin (hCG) followed by timed intercourse.

In case of IUI, semen samples will be processed within one hour of ejaculation by density gradient centrifugation followed by washing with culture medium. Women will be inseminated 36 hours after hCG injection. IUI will be performed once per cycle.

### Follow up

Pregnant women will undergo an ultrasound at 7 and 11 weeks of gestation to classify the pregnancy as clinical or ongoing singleton or multiple pregnancy.

Women will be contacted by telephone to enquire on the pregnancy, delivery and the health of the child. Detailed information on maternal complication will be obtained from the obstetrician treating the woman concerned. The focus will be on gestational diabetes and hypertensive disorders. When necessary, child health centres and/or paediatricians will then be contacted for specific information. Certainly not all couples will complete the 8 months of treatment. Drop-outs will largely represent normal patient flow. We aim to keep track of all drop-outs and to document the reason for the drop-out in the database.

A structured case record form (CRF) is used to register reproductive outcome, fertility treatments as well as the course and outcome of subsequent pregnancies.

### Withdrawal of individual patients

Women can leave the study at any time for any reason if they wish to do so. Women who drop out of the study will be asked to provide the reason for dropping out. This reason will be recorded. Women who drop out of the study will be treated according to the local protocols and guidelines.

### Outcome measures

#### Primary outcome measure

The main primary outcome measure will be birth of a healthy child resulting from a pregnancy that was established in the first eight months after randomisation. A live birth is defined as any ongoing pregnancy with a gestation time beyond 24 weeks. After randomisation women will receive a maximum of six treatment cycles.

#### Secondary outcome measures

• Multiple pregnancy, defined as a registered heart beat of at least two fetuses at 12 weeks of gestation.

• Clinical pregnancy, defined as any registered heart beat at sonography.

• Miscarriage, defined as loss of an intra uterine pregnancy (confirmed by ultrasound or histological examination) before the 20th week of pregnancy.

• Ovulation rates, assessed by a biphasic basal temperature curve, midluteal progesterone (> 16 nmol/l), detection of LH surge or sonography.

#### Background and demographic characteristics

To assess whether the treatment groups are balanced, the study populations will be compared for baseline measurements including female age, type of infertility (primary/secondary), duration of infertility, intoxications, body mass index, as well as sperm analysis according to WHO standards.

### Analysis

The analysis of all outcomes is on an intention to treat basis. Couples will be treated within a time horizon of eight months. Two comparisons will be made, one in which CC is compared to gonadotrophins and one in which the addition of IUI is compared to ovulation induction only. Differences in pregnancy outcomes will be expressed in relative risks with corresponding 95% confidence intervals. Birth rates over time will be compared using life tables. Discrete variables (e.g. presence of absence of a complication) will be summarised by frequencies and proportions. Differences between groups with respect to discrete variables will be evaluated by using chi-squared tests. Continuous variables will be assessed for normality and equality of variance between groups. For continuous variables, analysis of variance and/or regression will be used where appropriate.

#### Economic evaluation

The economic evaluation will be performed alongside the clinical trial. The economic evaluation will be designed as a cost-effectiveness analysis of the addition of IUI, use of gonadotrophins or the combination of both as compared to CC alone, with the costs per singleton pregnancy within eight months after randomisation resulting in live birth as the primary outcome measure. We will also assess the time to a successful pregnancy in the cost-effectiveness analysis. In this analysis, costs and effects will be discounted for 4% per year.

### Determining costs

The economic analysis will be performed from a societal perspective. A distinction will be made between costs of medical interventions (direct costs) and costs resulting from productivity losses (indirect or time costs). Standardised unit costs will be calculated for all centres based on actual expenses made during the study. Subsequently, unit costs will be applied to resource use as observed in participating centres. Resource utilisation will be documented using individual patient data in the CRF’s. Detailed information on complications will be obtained. Resource unit prices will reflect the unit of staff, materials, equipment, housing, depreciation and overhead. Productivity loss will be valued using Dutch reference data (hand book of the Dutch health council). Costs will be presented in Euros.

For each cost category, costs are measured as the volumes of resources used multiplied with appropriate valuations (unit-costs). Unit costs will be estimated according to the Dutch guideline on (unit) costing in health care (Oostenbrink, Bouwmans etal., 2004). Visits to GPs, medical specialists and paramedical care, and travelling will be valued based on the guideline prices. The friction cost method will be used to estimate the duration of lost productivity, age adjusted average daily wages will be used to value this duration. An analysis based on reimbursement fees is added (insurer perspective). Study-specific costs are excluded from analysis.

### Cost-effectiveness analysis

Cost-effectiveness of each strategy will be expressed as costs per live birth. The incremental cost-effectiveness ratio (ICER), reflecting the extra costs required to obtain one additional live birth of each individualized treatment option compared to CC alone will be estimated as the ratio between difference in costs between strategies and the difference in pregnancy rates. A decision model will be used to evaluate the optimal strategy when also taking into account differences in multiple pregnancies, where the relative importance of different outcomes can be varied in order to evaluate whether this affects the results.

Robustness of the results (costs and health outcomes) for various assumptions and parameter estimates will be explored in sensitivity analyses and visualized in ICER-graphs and cost-effectiveness acceptability curves. In the economic analyses, we will also explore differences in patient characteristics (e.g. age, parity, ethnicity), as both costs as well as the treatment effect might differ between various groups.

### Power calculation

The analysis will be by intention to treat. Two comparisons will be made, one in which CC is compared to gonadotrophins and one in which the addition of IUI is compared to ovulation induction only. Assuming a live birth rate of 40% after CC, 55% after addition of IUI and 55% after ovulation induction with gonadotrophins, with an alpha of 5% and a power of 80%, we need to recruit 200 women per arm (800 women in total).

An independent Data and Safety Monitoring Committee has criticized the data of the first 150 women and concluded that a sample size re-estimation should be performed after including 320 patients (i.e. 80 per arm).

## Discussion

Subfertility occurs in 15,000 new couples in The Netherlands each year. In one-third of cases (about 5,000 women) this is due to anovulation because of WHO type II anovulation or PCOS. We estimate that annually 1,500 to 2,000 women will not have conceived after six cycles of CC, despite regular ovulation.

For women ovulating on CC but failing to conceive, no consensus exists on the optimal policy. All trials, randomised or not, have been directed at therapy naïve women, or at women with WHO type II anovulation or PCOS who remained anovulatory after CC, also called CC-resistant women. Women with who actually do ovulate on CC but do not conceive represent a larger group than CC-resistant women but have never been the focus of clinical trials. Guidelines do not indicate the optimal duration of treatment because randomised controlled trials have not been performed. As a consequence, there is large practice variation on the subject. Whereas some clinicians switch to ovulation induction with gonadotrophins with or without IUI, others continue CC treatment with IUI instead of intercourse.

There are theoretically 4 treatment options that differ in working mechanism, costs and treatment burden.

IUI is an alternative to intercourse, because it bypasses the cervical barrier, optimises timing and shortens the distance from spermatozoa to oocyt. However, IUI is much more expensive than intercourse; because sperm needs to be processed and extra hospital visits are needed. Compared to ovulation induction with clomiphene citrate, gonadotrophins require frequent subcutaneous injections and frequent clinical visits due to necessarity of close sonographic monitoring, and carry a higher risk of multiple gestations. Ovulation induction with CC will on average be about 750 euro less expensive than ovulation induction with gonadotrophins.

Discontinuing CC and starting ovulation induction with gonadotrophins and/or IUI should only be done if the probability of conception is improved or the time to conception reduced. There is a strong need for a large, randomised study that compares costs and effects of CC and gonadotrophins with or without IUI. The results of the M-OVIN study will help to make evidence-based guidelines for treatment in women with WHO type II anovulation with CC-failure.

## Competing interests

The authors declare that they have no competing interests.

## Authors’ contributions

MJN and NW are responsible for the overall logistical aspects of the trial. MJN drafted the paper. FvdV, MW, PH, GO, MJN and BWM designed the trial, were responsible for the development of the protocol, applied for a grant and have overall responsibility for the trial. All authors are responsible for implementation of the study and inclusion of the eligible women. All authors read and approved the final paper.

## Authors’ information

Participating hospitals, local trial coordinators:

Academic Medical Centre, Amsterdam;

M.J. Nahuis, MD, N.S. Weiss, MD, FvdVeen, MD, PhD, B.W.Mol, MD, PhD,

M.van Wely PhD

VU University Medical Centre, Amsterdam;

P.G.A. Hompes, MD, PhD, CB Lambalk, MD, PhD

St Antonius Hospital Nieuwegein; G.J.E. Oosterhuis, MD, PhD

University Medical Centre Nijmegen; K. Fleischer, MD, PhD,

University Medical Centre Rotterdam; J.S.E. Laven, MD PhD, LCM Nekrui

University Medical Centre Maastricht; R.J.T. van Golde, MD PhD

Universtiy Medical Centre Utrecht; A.J Goverde, MD, PhD

University Medical Centre Leiden; N.F. Klijn, MD

Isala Clinics Zwolle; BJ Cohlen, MD, PhD

Máxima Medical Centre, Veldhoven; C.A.M. Koks, MD, PhD

Medical Spectrum Twente, Enschede, D.A. Hoozemans, MD

Martini Hospital Groningen, M.H. Gerards, MD

Twee Steden Hospital; IAJ van Rooij, MD, PhD

St Elisabeth Hospital Tilburg; JMJ Smeenk, MD, PhD.

## Pre-publication history

The pre-publication history for this paper can be accessed here:

http://www.biomedcentral.com/1472-6874/13/42/prepub

## References

[B1] The Rotterdam ESHRE/ASRM-Sponsored PCOS consensus workshop group Revised 2003 consensus on diagnostic criteria and long-term health risks related to polycystic ovary syndrome Fertil Steril 2004 81 19 25 10.1016/j.fertnstert.2003.10.004 14711538

[B2] Kousta E White DM Franks S Modern use of clomiphene citrate in induction of ovulation Hum Reprod Update 1997 3 359 365 10.1093/humupd/3.4.359 9459281

[B3] Kafy S Tulandi T New advances in ovulation induction Curr Opin Obstet Gynecol 2007 19 248 252 10.1097/GCO.0b013e3280c60c9a 17495641

[B4] Brown J Farquhar C Beck J Boothroyd C Hughes E Clomiphene and anti-oestrogens for ovulation induction in PCOS Cochrane Database Syst Rev 2009 Issue 4 10.1002/14651858.CD002249.pub4 19821295

[B5] Cudmore DW Tupper WRC Induction of ovulation with clomiphene citrate Fertil Steril 1966 17 363 373 4952094 10.1016/s0015-0282(16)35947-7

[B6] Garcia CR Freeman EW Rickels K Behavioral and emotional factors and treatment responses in a study of anovulatory infertile women Fert Steril 1985 44 478 483 10.1016/s0015-0282(16)48915-6 4054319

[B7] Johnson JE Cohen MR Goldferb AF The efficacy of clomiphene citrate for induction of ovulation Int J Fertil 1966 11 265 270 5338648

[B8] Moll E Korevaar JC Bossuyt PM van der Veen F Does adding metformin to clomifene citrate lead to higher pregnancy rates in a subset of women with polycystic ovary syndrome? Hum Reprod 2008 23 1830 1834 10.1093/humrep/den182 18487613 PMC2474667

[B9] Gysler M March CM Mishell DR Jr Bailey E A decade’s experience with an individualized clomiphene treatment regimen including its effect on the postcoital test Fertil Steril 1982 37 161 167 7060766 10.1016/s0015-0282(16)46033-4

[B10] Neveu N Granger L StMichel P Lavoie HB Comparison of clomiphene citrate, metformin, or the combination of both for first-line ovulation induction and achievement of pregnancy in 154 women with polycystic ovary syndrome Fertil Steril 2007 87 113 120 10.1016/j.fertnstert.2006.05.069 17081535

[B11] Badawy A Allam A Abulatta M Extending clomiphene treatment in clomiphene-resistant women with PCOS: a randomized controlled trial Reprod BioMed Online 2008 16 825 829 10.1016/S1472-6483(10)60148-4 18549692

[B12] López E Gunby J Daya S Parrilla JJ Abad L Balasch J Ovulation induction in women with polycystic ovary syndrome: randomized trial of clomiphene citrate versus low-dose recombinant FSH as first line therapy Reprod Biomed Online 2004 9 382 390 10.1016/S1472-6483(10)61273-4 15511336

[B13] Homburg Clomiphene citrate – end of an era? A mini review Human reproduction 2005 20 2043 2051 10.1093/humrep/dei042 15878925

[B14] van Wely M Bayram N van der Veen F Bossuyt PM An economic comparison of a laparoscopic electrocautery strategy and ovulation induction with recombinant FSH in women with clomiphene citrate-resistant polycystic ovary syndrome Hum Reprod 2004 19 1741 1745 10.1093/humrep/deh319 15166128

[B15] Van Wely M Bayram N Van Der Veen F Bossuyt PM Predicting ongoing pregnancy following ovulation induction with recombinant FSH in women with polycystic ovary syndrome Hum Reprod 2005 1827 1832 15831514 10.1093/humrep/deh891

[B16] Bayram N van Wely M Kaaijk EM Bossuyt PM van der Veen F Using an electrocautery strategy or recombinant follicle stimulating hormone to induce ovulation in polycystic ovary syndrome: randomised controlled trial BMJ 2004 328 192 10.1136/bmj.328.7433.192 14739186 PMC318481

[B17] Bayram N van Wely M van der Veen F Bossuyt PM Nieuwkerk P Treatment preferences and trade-offs for ovulation induction in clomiphene citrate-resistant patients with polycystic ovary syndrome Fertil Steril 2005 84 420 425 10.1016/j.fertnstert.2005.02.026 16084884

[B18] Moll E Bossuyt PM Korevaar JC Lambalk CB van der Veen F Effect of clomifene citrate plus metformin and clomifene citrate plus placebo on induction of ovulation in women with newly diagnosed polycystic ovary syndrome: randomised double blind clinical trial BMJ 2006 4 1485 16769748 10.1136/bmj.38867.631551.55 PMC1482338

[B19] Hammond MG Halme JK Talbert LM Factors affecting the pregnancy rate in clomiphene citrate induction of ovulation Obstet Gynecol 1983 62 196 202 6866363

[B20] Rossing MA Daling JR Weiss NS Moore DE Self SG Ovarian tumors in a cohort of infertile women N Engl J Med 1994 331 771 776 10.1056/NEJM199409223311204 8065405

[B21] Randall JM Templeton A Transvaginal sonographic assessment of follicular and endometrial growth in spontaneous and clomiphene citrate cycles Fertil Steril 1991 56 208 212 2070849 10.1016/s0015-0282(16)54473-2

[B22] Dickey RP Olar TT Taylor SN Curole DN Matulich EM Relationship of endometrial thickness and pattern to fecundity in ovulation induction cycles: effect of clomiphene citrate alone and with human menopausal gonadotropin Fertil Steril 1993 59 756 760 8458492 10.1016/s0015-0282(16)55855-5

[B23] Kolibianakis EM Zikopoulos KA Fatemi HM Osmanagaoglu K Evenpoel J Van Steirteghem A Endometrial thickness cannot predict ongoing pregnancy achievement in cycles stimulated with clomiphene citrate for intrauterine insemination Reprod Biomed Online 2004 8 115 118 10.1016/S1472-6483(10)60505-6 14759299

[B24] Thessaloniki ESHRE/ASRM-Sponsored PCOS Consensus Workshop Group Consensus on infertility treatment related to polycystic ovary syndrome Fertil Steril 2008 89 505 522 18243179 10.1016/j.fertnstert.2007.09.041

[B25] Bayram N van Wely M van der Veen F Recombinant versus urinary gonadotrophins or recombinant FSH for ovulation induction in subfertility associated with polycystic ovary syndrome Cochrane Database Syst Rev 2001 10.1002/14651858.CD002121 11406034

[B26] Guzick DS Ovulation induction management of PCOS Clin Obstet Gynecol 2007 50 255 267 10.1097/GRF.0b013e31802f361e 17304040

[B27] Nice guideline. Seehttp://www.nice.org.uk/nicemedia/live/10936/29269/29269.pdf

